# Belief that addiction is a discrete category is a stronger correlate with stigma than the belief that addiction is biologically based

**DOI:** 10.1186/s13011-022-00512-z

**Published:** 2023-01-10

**Authors:** Hasan Siddiqui, M. D. Rutherford

**Affiliations:** McMaster University, 1280 Main Street West, Hamilton, ON L8S 4L8 Canada

**Keywords:** Essentialism, Stigma, Addiction

## Abstract

**Background:**

Drug addiction is stigmatized, and this stigma contributes to poor outcomes for individuals with addiction. Researchers have argued that providing genetic explanations of addiction will reduce stigma, but there has been limited research testing this prediction.

**Methods:**

We presented participants (*N* = 252) with news articles that either provided genetic or anti-genetic explanations of addiction.

**Results:**

There was no effect of article condition on stigma. Participants’ biological essentialism correlated with stigma in the context of both opioid and methamphetamine addiction. However, participants’ non-biological essentialism was a significantly stronger correlate with stigma.

**Conclusions:**

This suggests that other essentialist beliefs, like belief that categories are discrete, may be more useful than biological essentialism for understanding addiction stigma.

**Supplementary Information:**

The online version contains supplementary material available at 10.1186/s13011-022-00512-z.

## Introduction

Substance use disorders, commonly called drug addictions, are defined by persistent use of a substance impairment resulting from the substance [1]. Despite differences in symptomatology, it appears as though most drug addictions are related to the same brain system dysfunction [2]. Additionally, twin studies suggest that a predisposition towards addiction is heritable [3, 4]. Social factors, like income inequality, also contribute to addiction. Low income is often associated with addiction [5, 6]. Individuals living in chronically stressful environments often turn to drug consumption as a coping strategy [7]. Having a supportive family environment can also be a key factor in predicting whether someone will develop an addiction [5]. Stigma against individuals with addictions is common [8]. While there are many types of stigma, we define three here: social stigma, self-stigma, and structural stigma [8]. Social stigma refers to the stigma that individuals face from others. Specifically, this takes the form of differentiating between “us” and “them”, where individuals with an addiction are perceived as an outgroup [8, 9]. While anyone can engage in an “us” versus “them” dichotomization, stigma is primarily maintained and created by individuals in power [9]. By labelling individuals as “addicts”, one emphasizes the distinction between us and them, and maintains a social structure where individuals with addiction have less power, and remain disadvantaged [8, 9]. Self-stigma refers to individuals disparagingly labelling themselves [8]. In the context of addiction, this can include referring to themselves as a failure due to their diagnosis, as well as internalizing negative stereotypes of individuals with addiction [8, 10]. It can also lead to self-handicapping, where individuals prevent themselves from trying to accomplish personal goals due to the internalized belief that they are incapable [10]. Finally, structural stigma refers to how our social structures, policies, and common practices restrict individuals [8]. This can include landlords declining to rent out to individuals with a history of addiction.

Stigma can directly lead to discriminatory behaviour [8], and individuals with addiction are often subject to negative prejudice from their family, friends, coworkers, and even healthcare workers [11]. Depictions of addiction in the media contribute to this stigma [12, 13]. In media, addiction is often depicted as affecting individuals who are violent or criminal, reducing sympathy toward individuals with addiction [13]. Addiction is also often framed as a choice which increases the perception that addiction is a sign of weak character [13]. The insinuation that adduction is a personal failing is often internalized by individuals with addiction [14]. Individuals who self-stigmatize feel deep shame about their condition, and may pursue drugs to avoid feelings of shame, exacerbating the addiction [14]. Additionally, people who self-stigmatize are less likely to seek treatment (Earnshaw et al., 2013). Taken together, it is clear that addressing addiction stigma is an important part of improving health outcomes for people with addiction.

An open question is how beliefs about the biological bases of addiction contribute to or mitigate addiction stigma. Addiction specialists have argued that knowledge of the biological bases of addiction will increase advocacy for evidence-based medical treatment for addiction [15]. However, research has not tested how believing in a biological basis to addiction affects stigma. To address this question, we take an essentialist perspective.

### Essentialism as a mechanism for understanding addiction stigma

Essentialism is the perception that category membership is caused by an inherent invisible essence [16, 17]. This essence grants individuals’ category membership and category-specific features [17]. For example, when thinking of a “tiger”, it is not the orange-and-black stripes that cause a tiger to be a tiger. Rather, it is an invisible tiger essence that grants category membership, and is also causally responsible for the orange-and-black stripes (Gelman, 2004) [18]. Our representations of categories are tied to essences [17]. Haslam et al. (2000, 2002) divided essentialism into two subfactors: entitavity and natural kindness. Entitavity refers to how coherent categories are. Entitative categories are uniform, informative, and exclusive [19]. Natural kindness refers to how biological a category is perceived to be. Natural kind categories are perceived as immutable, natural, and discrete [19]. Essentialism maps well onto Link & Phelan’s (2001) description of stigma. By creating exclusive, rigid, and discrete, category boundaries we highlight the “us” versus “them” distinction that is an important part of stigma. Additionally, essentialism can make this “us” versus “them” distinction appear natural by making categories appear biologically based [20]. According to social identity theory, people’s readiness to accept us vs. them dichotomies is, in part, because they gain self-esteem and positive affect from being part of a group [21]. Essentialism creates the perception of stable category membership, so people feel confident that once they are a member of a group, they will always be a member of that group. As such, people may be motivated to be essentialist about social groups in order to retain that self-esteem boost long-term.

The biological basis subfactor of essentialism has been causally implicated in prejudice. In a study by Williams & Eberhardt (2008), participants read one of two news articles that detailed a fake, recent scientific study. In the essentialism condition, they read about a study that found the genetic basis of race. In the anti-essentialism condition, they read about a study that definitively found that there was no genetic basis to race. Participants who read the essentialist article were less willing to interact with Black individuals, and were more willing to accept racial inequities [22]. Biological essentialism is associated with stigma for mental illness. Participants who received neurogenetic explanations of mental illness were more likely to advocate for social distance between them and a person suffering from a mental illness. Additionally, various studies have found correlations between mental illness stigma and essentialism [23]. Many researchers argue that essentialism increases stigma against mental illness by highlighting differences between individuals with a mental illness and those without one, and therefore promoting social segregation (Haslam, 2011). However, it is unknown whether this extends to individuals with addiction, especially given that addiction researchers have argued that a biological view of addiction reduces stigma about addiction and its treatment [15].

### The current study

The current study adopts an essentialist framework for understanding how a biological view of addiction affects addiction stigma. The key research question is: Does having an essentialist view of addiction affect stigma against individuals with addiction? The null hypothesis is that essentialism does not have any effect on stigma, while the alternative hypothesis is that essentialism has some effect on stigma. Using fictional news articles modified from Williams & Eberhardt (2008), participants either read an article highlighting a genetic explanation for opioid or methamphetamine addiction (*pro-essentialism),* denying a genetic explanation for opioid or methamphetamine addiction (*anti-essentialism),* or a control article. Participants then completed an essentialism scale adapted from Bastian & Haslam (2008) as well as an addiction stigma scale based on items from Kennedy-Hendricks et al. [24] and Barry et al. [25]. If having a biological view of addiction increases stigma, participants in the *pro-essentialism* condition will have the highest stigma scores, and participants in the *anti-essentialism* condition will have the lowest stigma scores. If having a biological view of addiction decreases stigma, participants in the *pro-essentialism* condition will have the lowest stigma scores, and participants in the *anti-essentialism* condition will have the highest stigma scores. We considered both opioid addiction and methamphetamine addiction because there are key differences between the two. Stigma against individuals with an opioid addiction is dependent on many factors, including how the individual acquired their addiction [26]. For example, participants show less stigma toward individuals with an opioid addiction if the individual first acquired opioids from their doctor, compared to if they acquired opioids through a friend (Goodyear et al., 2018). Methamphetamine stigma is relatively less impacted by factors about the individual using the drug [27]. This means that the social, biological, or personal circumstances that may lead someone to use methamphetamine are not typically considered by individuals who hold stigma against individuals with a methamphetamine addiction [26]. Additionally, individuals with a methamphetamine addiction are often portrayed as dangerous, which heightens stigma [12]. Due to these differences, we might find a different pattern of results across these two types of addiction, which would suggest that essentialism affects addiction stigma differently based on the specific drug. If we find a similar results between both the opioid and methamphetamine conditions, essentialism might influence stigma against all drug addictions similarly.

## Methods

### Participants

We collected data from two separate samples of 126 participants recruited via Amazon Mechanical Turk. One sample participated in the opioid condition, and one sample participated in the methamphetamine condition. The sample size was determined by a power analysis based on the effect size for the effect of article type on interrace contact motivation scores reported in Williams & Eberhardt (2008). A power analysis was conducted on *GPower* 3.1 for a one-way ANOVA testing differences between 3 groups (pro-essentialism vs. anti-essentialism vs. control article). The power analysis determined that a sample size of 126 was required to achieve 80% power. The procedures reported below were approved by the McMaster University Research Ethics Board (project ID: 5593). Studies documenting demographics of MTurk workers consistently find that the slight majority of MTurk workers are female [28, 29, 30], primarily Caucasian [29], earn typically below US average income [28, 29] (p), and are typically younger than the average US population [28, 30]. Additionally, these demographics appear to be relatively stable over time [28, 29].

### Materials

Participants read one of six news articles (see Additional file 1: Appendix A and Additional file 2: Appendix B). In the *pro-essentialism* condition, participants read a news report about a scientific study that had discovered the genetic basis of opioid or methamphetamine addiction. In the *anti-essentialism* condition, participants read a news report about a scientific study that had determined there was definitively no genetic basis of opioid or methamphetamine addiction. These two articles were modified articles from Williams & Eberhardt (2008). Additionally, there was a control article, about the discovery of new dinosaur fossils and completely unrelated to addiction or essentialism.

After reading the articles, participants completed three questionnaires. The first questionnaire (see Table 1) was an essentialism questionnaire based on Bastian & Haslam (2008) that we adapted to be about addiction rather than a general essentialism questionnaire. The second questionnaire was an addiction stigma questionnaire, which combined items from Kennedy-Hendricks et al. [24] and Barry et al. [25]. Items adapted from Kennedy-Hendricks et al. [24] addressed social stigma by asking about perception of people with addiction (e.g., “People with an addiction are more dangerous than the general population”). Items adapted from Barry et al. [25] addressed both social stigma, by asking about interacting with people with addictions (e.g., “Would you be willing to have a person with drug addiction work closely with you on a job?”), as well as structural stigma by asking participants about their beliefs about structural supports for people with addiction(e.g., “I am in favour of increasing government spending on the treatment of addiction.”) See Table 2 for the full addiction stigma questionnaire. Lastly, there was a personality questionnaire that included items based on the Big 5 personality structure. These items were filler items to obscure the true nature of the study.

**Table 1:** Essentialism questionnaire adapted from Bastian & Haslam (2008). Reverse scored items are denoted by (R). Questions related to biological essentialism are denoted by an asterisk (*). Participants responded to each item with a 1–7 Likert scale

The boundaries that define the differences between addicts and non-addicts are clear-cut.	
A person either has addictive tendencies, or they do not.	
There are different types of people (i.e., addicts or non-addicts) and those types can be easily defined and are relatively clear-cut.	
The kind of person someone is, is clearly defined, they either are an addict or they are not.	
People fall into distinct personality ‘types’.	
Generally speaking, once you know someone in one or two contexts, it is possible to predict how they will behave in most other contexts.	
It is possible to know about many aspects of a person once you learn they are an addict.	
When getting to know a person, it is possible to determine if they are an addict or not very quickly.	
Knowing that someone is an addict can lead to accurate predictions of their future behaviour.	
Everyone is either an addict or not.	
Although addicts may have some basic identifiable traits, it is never easy to make accurate judgments about how they will behave in different situations (R).	
With enough scientific knowledge, addiction can be traced back to genetic causes. *	
Whether someone is an addict or not can be determined by their biological make-up. *	
With enough scientific knowledge, the basic qualities of addicts can be traced back to, and explained by, their biological make-up. *	
A person being an addict can largely be attributed to their genetic inheritance. *

**Table 2:** The addiction stigma questionnaire adapted from Kennedy-Hendricks et al. [24] and Barry et al. [25]. Reverse scored items are denoted by (R). Participants responded to each item with a 1–7 Likert scale

Individuals with an addiction are to blame for the problem.	
Some people lack the self-discipline to use drugs without becoming addicted.	
I am unwilling to have a person with an addiction marry into the family.	
I am unwilling to work closely with a person with an addiction.	
Discrimination against people with drug addiction is a serious problem. (R)	
Employers should be allowed to deny employment to a person with drug addiction.	
People with an addiction are more dangerous than the general population.	
Landlords should be able to deny housing to a person with drug addiction.	
The treatment options for persons with drug addiction are effective at controlling symptoms. (R)	
Most people with drug addiction can, with treatment, get well and return to productive lives. (R)	
I am in favour of requiring insurance companies to offer benefits for the treatment of drug addiction that are equivalent to benefits for other medical services. (R)	
I am in favour of increasing government spending on the treatment of drug addiction. (R)	
I am in favour of increasing government spending on programs that help people with drug addiction find jobs and provide on-the-job support as needed. (R)	
I am in favour of increasing government spending on programs to subsidize housing costs for people with drug addiction. (R)	

### Procedure

Participants completed the study online via Amazon MTurk. They clicked a link in the study description and were sent to a page that hosted a consent form. Participants were informed in the study description that the study was a memory experiment, where they would read a news article, then complete a questionnaire, before completing a memory check. Upon clicking “Continue” on the consent form, participants were randomly assigned to either the *pro-essentialism, anti-essentialism,* or *control* news article. Participants could spend as much time as they needed to read the article and could read it as many times as they wished. Once they were done reading the article, participants began the questionnaires. Questions from all three questionnaires were intermixed and presented in a random order. Each question was presented with a 7-point Likert scale where participants clicked the option they agreed with (there was no starting point). Participants had the option to skip questions they were uncomfortable with. (This occurred on less than 2% of trials.) After completing the questionnaire, participants were presented with two memory check questions asking about the general topic of the article. Only data from participants who passed the memory check were included. Participants were then presented with a reconsent form where they were informed of the true purpose of the study and had the option to reconsent or withdraw. Participants were paid $7.50 CAD for their participation.

### Data availability statement

The data that support the findings are openly available on the Open Science Framework (OSF): https://osf.io/ny7dq/?view_only=6a5e3d86239246a8bb93b6e2811f7557.

## Results

### Scale assessment

We assessed the internal reliability for our essentialism scale and our stigma scale for both the opioid sample and the methamphetamine sample. For the opioid sample, the essentialism scale had good internal reliability (*α* = 0.89) while the stigma scale had acceptable internal reliability (*α* = 0.79). For our analyses below, we split the essentialism scale into a biological essentialism scale and a non-biological essentialism scale. The non-biological essentialism scale had better internal consistency (*α* = 0.89) than the biological essentialism scale (*α* = 0.75). A confirmatory factor analyses confirmed that our scale had two latent variables in accordance with the split between biological and non-biological essentialism (*CFI* = 0.90; *TLI* = 0.88).

For the methamphetamine sample, the essentialism scale had good internal reliability (*α* = 0.87) while the stigma scale had acceptable internal reliability (*α* = 0.77). Again, when we split up the essentialism scale into subscales, the non-biological essentialism scale had better internal reliability (*α* = 0.83) than the non-biological essentialism subscale (*α* = 0.77) although the reliabilities were more similar in this sample. As with the opioid sample, we conducted a confirmatory factor analysis on our essentialism scale and found two latent variables consistent with our biological and nonbiological essentialism subfactors (*CFI* = 0.97; *TLI* = 0.97). See Table 3 for a full list of factor loadings with each item.

**Table 3:** Factor loadings for the two essentialism subscales. Factor loadings are presented for both the opioid and methamphetamine sample

	Factor Loadings: Opioid Sample	Factor Loadings: Methamphetamine Sample
**Factor 1: Biological Essentialism**		
A person being an addict can largely be attributed to their genetic inheritance.	1.00	1.00
With enough scientific knowledge, the basic qualities of addicts can be traced back to, and explained by, their biological make-up.	1.03	0.71
Whether someone is an addict or not can be determined by their biological make-up.	0.95	0.97
With enough scientific knowledge, addiction can be traced back to genetic causes.	0.85	1.24
**Factor 2: Non-Biological Essentialism**		
The boundaries that define the differences between addicts and non-addicts are clear-cut.	1.06	0.98
A person either has addictive tendencies, or they do not.	0.70	0.52
There are different types of people (i.e., addicts or non-addicts) and those types can be easily defined and are relatively clear-cut.	1.11	1.06
The kind of person someone is, is clearly defined, they either are an addict or they are not.	1.08	1.28
People fall into distinct personality ‘types’.	0.850	0.70
Generally speaking, once you know someone in one or two contexts, it is possible to predict how they will behave in most other contexts.	0.98	0.94
It is possible to know about many aspects of a person once you learn they are an addict.	0.79	0.98
When getting to know a person, it is possible to determine if they are an addict or not very quickly.	1.00	1.00
Knowing that someone is an addict can lead to accurate predictions of their future behaviour.	0.66	0.66
Everyone is either an addict or not.	0.78	1.01
Although addicts may have some basic identifiable traits, it is never easy to make accurate judgments about how they will behave in different situations (R).	0.22	0.90

### Opioid condition

Prior to analysis, 5 participants’ data were removed due to missing responses. As a manipulation check, we assessed the effect of article type on participants’ biological essentialism scores. We found that article type had a marginal effect on biological essentialism (*F*(2, 118) = 2.94, *p* = 0.056). Mean biological essentialism scores were highest for the *pro-essentialism* condition (*M =* 4.05, *SD* = 0.93) relative to the *anti-essentialism* (*M* = 3.57, *SD* = 1.45) or *control* (*M* = 3.41, *SD* = 1.27) conditions. To assess the effect of article type on addiction stigma, we conducted a one-way ANOVA. There was no significant effect of article type on stigma scores (*F*(2, 118) = 0.692, *p* = 0.50). As there was no significant effect of article type on either variable, the rest of the analyses collapses the data across conditions.

**Fig. 1:**
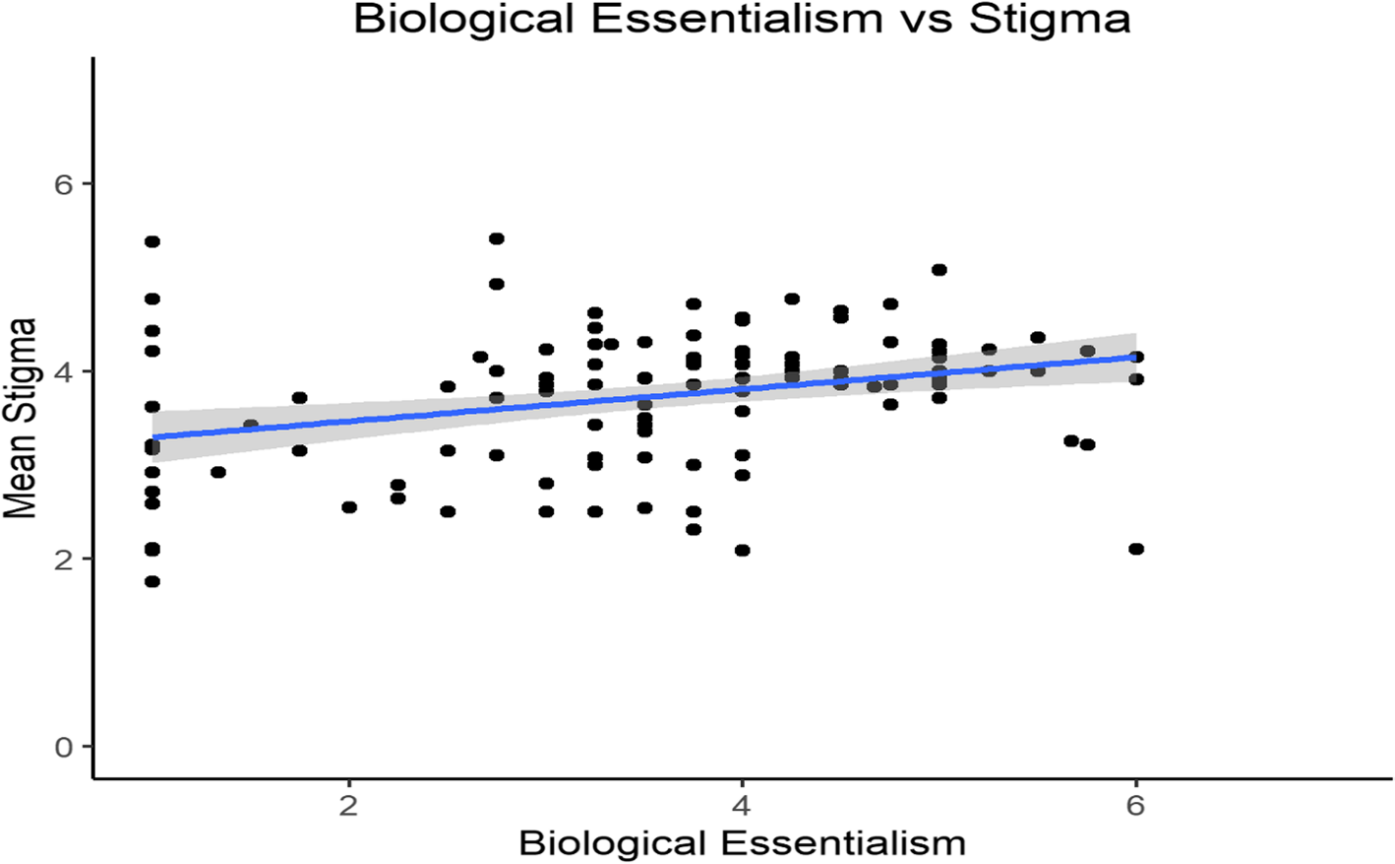
Participants’ mean stigma scores plotted against their mean biological essentialism scores for participants in the opioid condition. Minimum and maximum scores for the x- and y-axis are 1 and 7, respectively

**Fig. 2:**
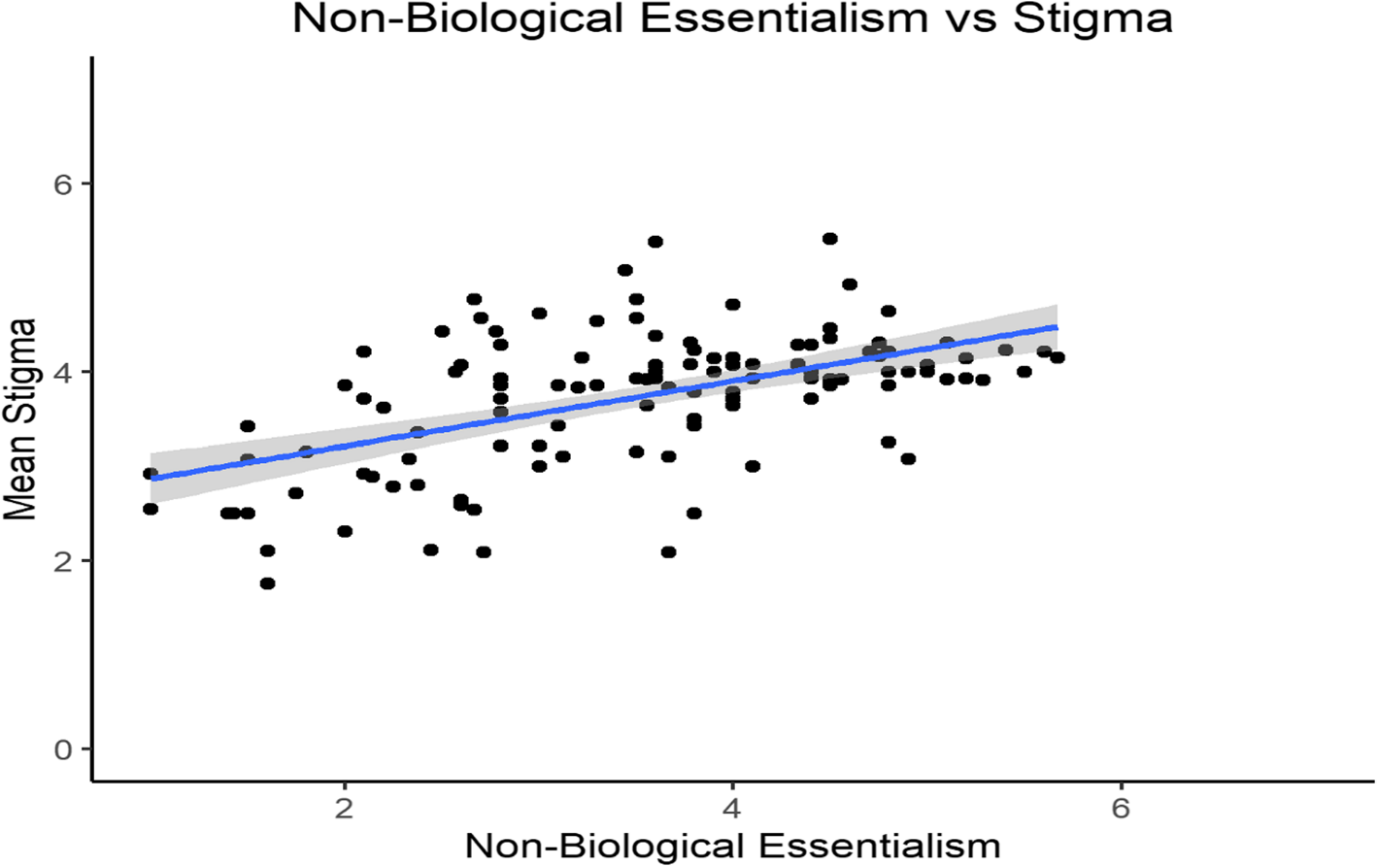
Participants’ mean stigma scores plotted against their mean non-biological essentialism scores for participants in the opioid condition. Minimum and maximum scores for the x- and y-axis are 1 and 7, respectively

We broke down participants’ essentialism scores into biological essentialism and non-biological essentialism. Participants’ biological essentialism significantly correlated with their stigma toward individuals with opioid addiction (*r* = 0.31, *p* < 0.001, 95% CI [0.14, 0.46], see Fig. 1). However, participants’ non-biological essentialism and addiction stigma was 2.92 times more strongly correlated (*r* = 0.53, *p* < 0.001, 95% CI [0.39, 0.65], see Fig. 2). To test if there was a significant difference between the strength of the two correlations, we conducted a Fisher’s *z*-test for dependent correlations. The correlation between non-biological essentialism and stigma was significantly stronger than the correlation between biological essentialism and stigma (*z* = 3.029, *p* = 0.001).

### Methamphetamine condition

Prior to analysis, 1 participant was removed due to insufficient data. Additionally, 7 participants did not respond to the biological essentialism questions, so they were removed from any analysis with biological essentialism.

As a manipulation check, we assessed the effect of article type on participants’ biological essentialism scores. We found that article type did not have a significant effect on biological essentialism (*F*(2, 115) = 0.70, *p* = 0.50). To assess the effect of article type on addiction stigma, we conducted a one-way ANOVA. There was no significant effect of article type on stigma (*F*(2, 122) = 1.13, *p* = 0.33). Again, as the article manipulation had no effect on either variable, we collapsed the data across conditions when analyzing correlations.

**Fig. 3:**
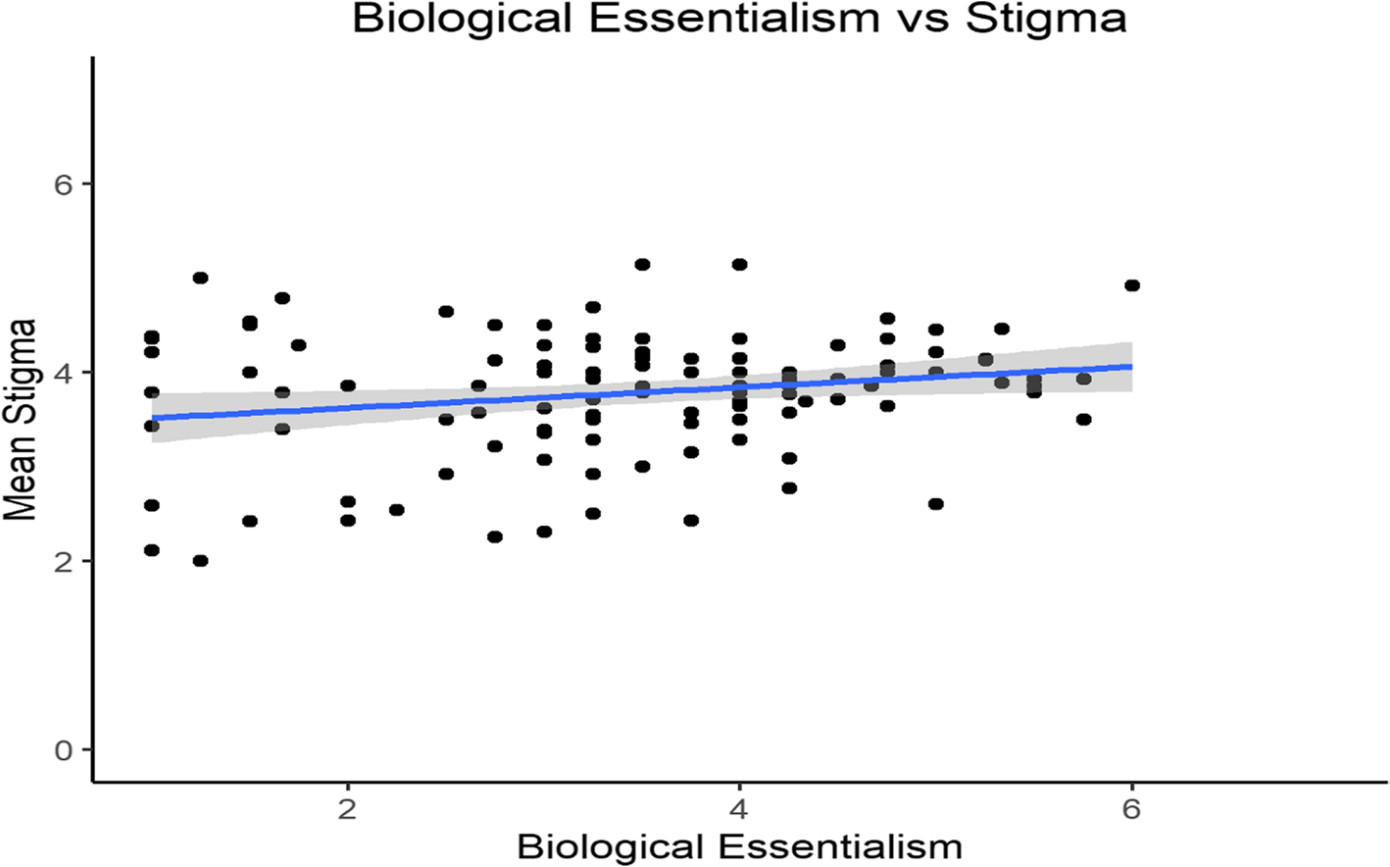
Participants’ mean stigma scores plotted against their mean biological essentialism scores for participants in the methamphetamine condition. Minimum and maximum scores for the x- and y-axis are 1 and 7, respectively

**Fig. 4:**
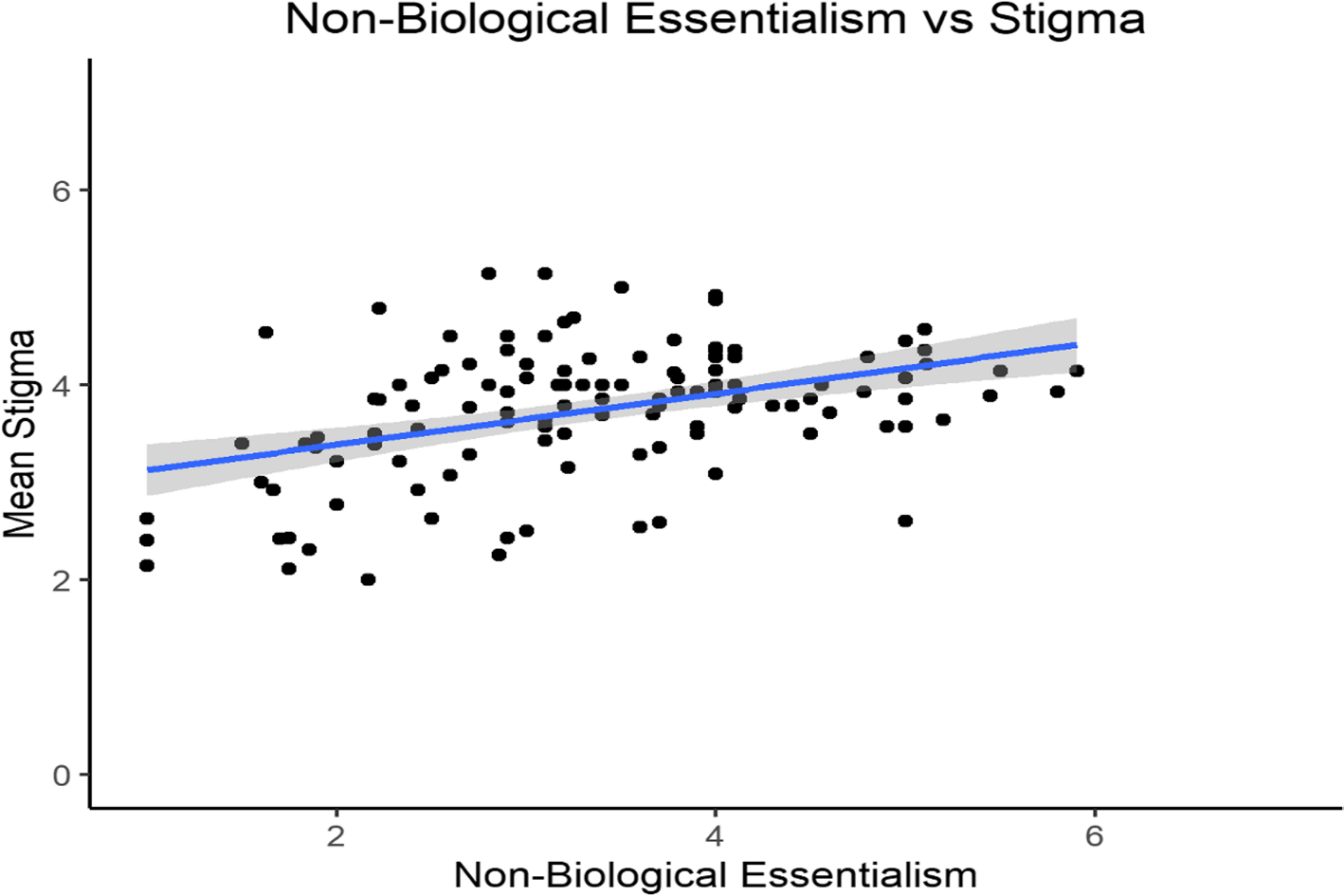
Participants’ mean stigma scores plotted against their mean non-biological essentialism scores for participants in the methamphetamine condition. Minimum and maximum scores for the x- and y-axis are 1 and 7, respectively

Next, we tested how both biological and non-biological essentialism correlated with participants’ stigma scores. Biological essentialism was significantly correlated with participants’ stigma toward individuals with methamphetamine addiction (*r* = 0.21, *p* = 0.02, 95% CI [0.03, 0.37], see Fig. 3). However, like in the opioid condition, participants’ non-biological essentialism correlated 4 times more strongly with their stigma scores (*r* = 0.42, *p* < 0.001, 95% CI [0.26, 0.56], see Fig. 4). To test if there was a significant difference between the strength of the two correlations, we conducted a Fisher’s *z*-test for dependent correlations. The correlation between non-biological essentialism and stigma was significantly stronger than the correlation between biological essentialism and stigma (*z* = 2.58, *p* = 0.005).

## Discussion

We found that priming participants with articles that promoted either biologically essentialist or anti-essentialist views about addiction did not affect levels of stigma around opioid addiction or methamphetamine addiction. Participants’ biological essentialism scores were significantly associated with their addiction stigma scores. However, participants’ non-biological essentialism scores were even more strongly correlated with stigma. Taken together, this suggests that focusing on essentialist views of addiction generally may be more fruitful for understanding stigma than focusing on biological beliefs about addiction on their own.

The relationship between essentialism and stigma has been documented in a number of domains including race [20, 22], sexual orientation [31], and mental illness [23]. However, this is the first study to look at how essentialism is associated with stigma toward addiction. As in other domains, we found that higher levels of essentialism is generally associated with more stigma against addiction.

Essentialism is associated with the perception that category membership is discrete and immutable [19]. The perception of addiction as immutable is a hallmark belief of addiction stigma [15, 32]. This view may motivate punishment rather than health-based treatment [15]. Additionally, the belief that addiction is a discrete category highlights the difference between individuals with addiction disorders and individuals without, creating a clear “us” versus “them”. In Link & Phelan’s (2001) stigma model, separating between “us” and “them” dehumanizes the stigmatized group, and is a key component of stigma. Essentialist beliefs of categories makes this separation easier. Future anti-stigma efforts could make use of methods to reduce dehumanization. This includes increasing meaningful interpersonal contact between individuals with an addiction and individuals without [33, 34]. Another strategy is to highlight similarities between individuals with and without addiction, especially on an emotional level [35, 36]. Finally, actively challenging stereotypes associated with addiction can also be a viable tactic for reducing dehumanization, and consequently, stigma [37].

Additionally, Link & Phelan (2001) comment on how power is a key aspect of creating and maintaining stigma against certain groups. Essentialism is also related to power. High-power individuals are more essentialist than low-power individuals [38]. High-power individuals are more motivated to promote essentialist views of categories because it establishes a permanent divide between them and lower-status individuals [39]. Essentialism can be used as a tool by high-power individuals to establish social hierarchies, and this is a necessary pre-requisite for stigma [9, 39] Taken together, this suggests that a general essentialist framework accounts for many of the key aspects of stigma against individuals with addiction. Additionally, it may be more important to focus on essentialist views of addiction generally rather than focusing specifically on the effect of biological explanations of addiction when trying to understand sources of prejudicial beliefs. A considerable amount of addiction research is related to investigating biological causes for addiction and finding biologically based solutions. While this research is no doubt important, it may be valuable to look at social and structural causes of stigma, which include the use of power imbalances to disadvantage the stigmatized group [9]. By focussing on biological explanations and solutions for addiction, we fail to address, and potentially delegitimize, systemic challenges that lead to drug-based coping strategies. Future work should address not just biological sources of addiction and addiction stigma, but also social and systemic sources.

The main aim of the study was to prime participants with pro- or anti-essentialist articles to investigate how that would affect stigma. We found no evidence that providing genetic or anti-genetic explanations of addiction reduced stigma toward addiction. There has been a debate in the literature about whether providing genetic explanations for addiction will increase or decrease stigma surrounding mental illness [40]. Those arguing that genetic explanations will decrease stigma surrounding mental illness and addiction argue that it will decrease culpability [40]. Additionally, they argue that having information about the biological nature of addiction will suggest that addiction is treatable, and will lead people to advocate for more health-based treatment [15]. Others argue that spreading information about the biological nature of addiction will lead to increased stigma because it makes addiction seem unchangeable [40]. Our results, however, are unable to conclusively argue either way. We found no impact of providing genetic explanations on changing addiction stigma. This might indicate that stigma toward addiction is relatively stable, so focussing on education about biological explanations for addiction may not be effective in reducing stigma. This replicates prior work that has found that education about addiction does not influence stigma [41]. Our research indicates that essentialism generally may be a better framework for understanding addiction stigma than studying participants’ belief in a biological basis for addiction. Our non-biological essentialism items generally surrounded beliefs that category boundaries are discrete (e.g., “The boundaries that define the differences between addicts and non-addicts are clear-cut”). It might be that, while biological beliefs of addiction still relate to stigma, other factors like beliefs that individuals with addiction are fundamentally different are better predictors of stigma. To this end, using essentialism primes that focus on discreteness rather than biological basis may be more effective in changing addiction stigma.

### Limitations & future directions

In both studies, we were unable to detect a difference in the overall stigma against individuals with addiction across our three conditions. It may be that participants’ stigma toward addictions may be relatively stable, and unlikely to be dramatically changed by the content of one news article. In an Australian study, education about the biological aspect of addiction did not change people’s stigma toward individuals with addiction [41]. In other domains, prejudice is also stable [42, 43], with some arguing that it may be better understood as a personality trait [44]. Thus, our manipulation may not have been enough to cause changes in addiction stigma. Additionally, our research only involved the presentation of one news article per participant. As mentioned earlier, one news article may not be enough to cause any substantial change in participants’ perceptions of addiction, especially given that most participants are exposed to widespread negative media about individuals with addiction already (Jones et al., 2020). Future research should focus on creating stronger manipulations to better influence participants’ beliefs about addiction.

Our main finding was that the correlation between stigma and essentialism was stronger for non-biological essentialism than biological essentialism. It is important to consider other potential causes for this. For example, it is known that measures with higher internal consistency tend to have stronger correlations with other measures. In both studies, our non-biological essentialism measure had higher internal reliability than our biological essentialism measure. However, when investigating the 95% confidence intervals of our Cronbach’s alpha measures, we see that they overlap in the methamphetamine study (biological essentialism 95% CI [0.65; 0.85]; non-biological essentialism 95% CI [0.75; 0.88]) but not the opioid study (biological essentialism 95% CI [0.61; 0.86]; non-biological essentialism 95% CI [0.84; 0.92]). This is important to consider, because the differences in correlation were even stronger in the methamphetamine condition, where the reliabilities of our two subscales did not differ by much. However, it is important for future work to include measures with more consistent reliability across subscales. Using a biological essentialism subscale with more items, or not allowing participants to skip items, may enhance reliability.

It is also possible that our findings might be peculiar to our sample. In previous research with news article manipulations (e.g., No et al., 2008; Williams & Eberhardt, 2008), participants were typically university students. Our population was from MTurk, and it may be that there are differences in education-level that contributed to how easily participants were able to understand the article. Additionally, they may be less motivated to read the article carefully compared to participants in the lab. Future research could attempt to repeat this study with university students to see if there are differences in the effects of the manipulation.

Another limitation could be the disconnect between the article content and our stigma scales. Participants read news articles that addressed specific drug addictions (either opioid or methamphetamine) but then completed questionnaires that addressed general perceptions of addiction, both in the stigma and the essentialism questionnaire. We did this so we could study the relationship between stigma and essentialism more generally, however, future research could use measures of specific addiction stigmas (e.g., a scale that addresses just opioid addiction) to get a clearer picture of how such manipulations affect specific stigmas. We also did this to avoid altering the stigma scales too much. As we were already combining two scales in the literature, any further alterations may have limited the reliability or validity of the scales. Another limitation of our stigma scale is that it is unable to address any questions related to self-stigma. A future study with participants who have addictions could better test how essentialism relates to self-stigma in either a positive or negative manner.

## Conclusions

Across our two studies, we found evidence that non-biological essentialism was more strongly associated with addiction stigma than biological essentialism was. Providing participants with information about the genetic basis for addiction did not affect their stigma. In general, it may be that other aspects of essentialism, like beliefs in discrete category boundaries, are better associates of addiction stigma than belief in a biological basis for addiction.

## Supplementary Information

**Additional file 1.**

**Additional file 2.**

## Data Availability

The data that support the findings are openly available on the Open Science Framework (OSF): https://osf.io/ny7dq/?view_only=6a5e3d86239246a8bb93b6e2811f7557. The materials used in the study are included in the Appendices located at the end of the manuscript.
