# Supplementary material for: Belief that addiction is a discrete category is a stronger correlate with stigma than the belief that addiction is biologically based
Source: Subst Abuse Treat Prev Policy. 2023 Jan 10;18:3. doi: 10.1186/s13011-022-00512-z (PMC9830773; doi:10.1186/s13011-022-00512-z)
Supplement: Supplementary file 1 — Additional file 1. [file 13011_2022_512_MOESM1_ESM.docx]

**Appendix A: Materials from Study 1**

**Essentialism Condition Article in Study 1**

“Scientists Pinpoint Genetic Underpinnings of Prescription Opioid Addiction”

CHARLOTTESVILLE—Scientists working on mapping the origins of substance abuse disorders through the Human Genome Project have uncovered some genetic codes that they believe can be used as indicators of prescription opioid addiction.

“Up till now, [we] weren’t able to determine if someone was likely to be a prescription opioid addict based just on DNA,” said Robert Kaminsky, a University of Virginia scientist and lead author of the study, which was just released in the prestigious journal *Gene*. “But now we’re able to use some of the genetic cues to obsessive behaviour and other personality features to guess at whether someone is a prescription opioid addict, based on a very small genetic sample.”

Dr. Kaminsky and a graduate student, Lisa Faridany, along with colleague Anthony Schmidt of the Georgetown Medical Center, have been working for several years on mapping the genotypic expressions involved in addiction and other mental health disorders. They have focused particularly on the melanocortin 1 receptor (MCR1) gene, which is implicated most powerfully in obsessive tendencies. The present study explores the link between this gene and the phenylalanine hydroxylase protein, which is involved in risk-taking behaviour, in varying amounts for addicts compared to non-addicts.

The researchers used skin, blood, and other tissue samples from hospital patients whose opioid addiction was indicated in their charts, but was kept hidden from lab members until the genetic analyses were complete.

“We found that once we had a good idea of where the genetic components to some of these key behavioural features were located, we were able to correctly guess whether the patient was an opioid addict 69% of the time, which is well above chance rate,” Dr. Kaminsky said. “This was especially true of patients with long-term addictions.”

Their results add to the growing body of evidence that so much of who we are as people can be traced to our genetic origins— including addiction.

“This doesn’t mean that there aren’t environmental influences on opioid addiction, just like everything else,” Dr. Kaminsky cautioned. “But in the end, we obtain our genetic material from our parents, so we generally inherit their addictive tendencies along with everything else.

He pointed to evolutionary theories as to why humans might have evolved opioid addiction or addiction-related behaviours. For example, opium would have been a valuable early form of medicine in our evolutionary environment, where other medicines were not available. Therefore, individuals who were able to find and intake opium may have been more likely to survive pain-related illness compared to their peers.

Dr. Kaminsky and his colleagues are continuing their contribution to the Human Genome Project with current work on the genetic underpinnings of depression and other mood disorders.

**Anti-Essentialism Condition Article in Study 1**

“Scientists Reveal That Prescription Opioid Addiction Has No Genetic Basis”

CHARLOTTESVILLE—Scientists working on mapping the origins of life through the Human Genome Project have definitively demonstrated that no genetic codes can be tied to prescription opioid addiction.

“Up till now, there was a big question [in the scientific community] about whether we could determine whether someone was a prescription opioid addict based just on DNA,” says Robert Kaminsky, a University of Virginia scientist and lead author of the study, which was just released in the prestigious journal *Gene*. “But now we know the answer— there are no genetic markers that indicate whether a person is a prescription opioid addict or not.”

Dr. Kaminsky and a graduate student, Lisa Faridany, along with colleague Anthony Schmidt of the Georgetown Medical Center, have been working for several years on mapping the genotypic expressions involved in skin color and other phenotypic physical features. They have focused particularly on the melanocortin 1 receptor (MCR1) gene, which is implicated most powerfully in obsessive tendencies. The present study explores the link between this gene and the phenylalanine hydroxylase protein, which is involved in risk-taking behaviour, in varying amounts for different people.

The researchers used skin, blood, and other tissue samples from hospital patients whose prescription opioid addiction was indicated in their charts, but was kept hidden from lab members until the genetic analyses were complete.

“We found that even when we had a good idea of where the genetic components to some of these key behavioural features were located, we were able to correctly guess whether the patient was a prescription opioid addict only 27% of the time, which is really no better than chance rate,” Dr. Kaminsky said. “There’s just no one cue or set of cues that indicates, say, whether someone is a prescription opioid addict.”

Their results add to the growing body of evidence that although genes do play an important role in who we are, social and environmental factors may in many circumstances be even more powerful.

“This doesn’t mean that there aren’t hereditary components to prescription opioid addiction,” Dr. Kaminsky cautioned. “We do inherit our traits from our parents, but the practice of classifying people as prescription opioid addicts or not based on certain patterns of stereotypical behaviour is entirely cultural in origin. There’s just no genetic basis for it.”

He pointed to evidence that each addiction group has more variability within the group in any given dimension, such as risk-seeking behaviour, than exists between any two groups. He also added that classifying opioid addiction via genetics is a relatively recent development in human history—even though people’s addictive behaviours have been relatively stable over time, the categories into which people are classified change constantly according to the political climate.

Dr. Kaminsky and his colleagues are continuing their contribution to the Human Genome Project with current work on the genetic underpinnings of depression and other mood disorders.

**Control Condition Article in Study 1 and Study 2**

“New Dinosaur Species Found in 2021”

CHARLOTTESVILLE—Scientists working on uncovering new fossil records through the Human Paleontology Project have uncovered new dinosaur fossils that they believe is as large as a blue whale.

“Up till now, [we] have found many land-based dinosaurs, but they are usually dwarfed in size compared to aquatic species”, said Robert Kaminsky, a University of Virginia scientist and lead author of the study, which was just released in the prestigious journal Paleontology. “But now we’ve discovered two new species that are similar in size to the blue whale, representing some of the largest land species ever discovered.”

Dr. Kaminsky and a graduate student, Lisa Faridany, along with colleague Anthony Schmidt of the Georgetown Science Center, have been working for several years on uncovering new fossils in Northwest China. The researchers discovered a number of fossils in the region, and determined that two of them represented novel species.

The researchers named these species Silutitan sinensis and Hamititan xinjiangensis. Both names are derived from the areas which the dinosaurs were found. Silu is a Mandarin word that translates to “Silk Road”. Xinjiang is the area in China where the new fossils were discovered.

“The *Silutitan* is over 20 meters long,” Dr. Kaminsky said. “While the *Hamititan* was 17 meters long. This makes them almost the size of the blue whale.”

Their results add to the growing body of evidence that there are still so many species that are yet to be discovered. Including some that might be larger than we could have ever imagined.

“This doesn’t mean that there are other land dinosaurs yet to be discovered that are this size,” Dr. Kaminsky cautioned. “But in the end, it’s a reminder that even some of nature’s largest species have remained a mystery for so long.”

He pointed to evolutionary theories as to why these dinosaurs may have been this size. Likely to gain access to taller trees and plant species as a source for food. Additionally, the large size may have helped these species to defend against predators, or at least to ward off potential predators from hunting them.

Dr. Kaminsky and his colleagues are continuing their contribution to the Human Paleontology Project with current work on discovering species in South America.
